# *BAG3*-related myofibrillar myopathy: focus on its cardiac involvement

**DOI:** 10.3389/fgene.2025.1636999

**Published:** 2025-11-26

**Authors:** Elise Daire, Elena Panaioli, Cyril Gitiaux, Catherine Gardin, Victor Waldmann, Damien Bonnet, Karim Wahbi, Diala Khraiche

**Affiliations:** 1 Pediatric Cardiology Department, Amiens University Hospital and Laboratory EA4666 Hematim, University of Picardie–Jules Verne, Amiens, France; 2 M3C-Necker, Congenital and Pediatric Cardiology Department, Necker Enfants malades University Hospital, APHP, Paris, France; 3 Reference Centre for Neuromuscular Diseases, Necker-Enfants malades Hospital, APHP, Paris, France; 4 Cardiology Departement, Laval Hospital, Laval, France; 5 University Paris Cité, Paris, France; 6 APHP, Cochin Hospital, Cardiology Department, Centre de Référence de Pathologie Neuromusculaire, Paris, France

**Keywords:** cardiomyopathy, pediatric, *BAG3*, Bcl-2-associated athanogene 3, myofibrillarmyopathy, restrictive cardiomyopathy

## Abstract

Myofibrillar myopathy is a cause of rare and severe pediatric cardiomyopathies. Few descriptions of patients carrying the rare p. Pro209Leu variant in *BAG3* and presenting with myofibrillar myopathy are reported in the literature. Most reports originate from neurological teams, while the cardiac phenotype remains poorly described, even though it is crucial for prognosis, as cardiac involvement can significantly influence patient outcomes. We focused on the cardiac phenotype associated with p. Pro209Leu variant in *BAG3* and conducted a literature review. We report three patients with severe restrictive cardiomyopathy (RCM) including two with left ventricular hypertrophy. Cardiac symptoms appeared 7 [5–7.5] years after neurological onset and were predominantly right heart failure, with high NT-proBNP levels, and arrhythmic events (atrial flutter, ectopic atrial tachycardia). Cardiac MRI showed biatrial and left ventricular fibrosis. Prognosis was severe, with two deaths. In the reviewed cases, cardiac involvement was present in 75%–77.3% of patients and was diagnosed at an early age. Restrictive cardiomyopathy was the most prevalent phenotype (68%–75%), whereas few patients exhibited hypertrophic cardiomyopathy or borderline QT interval. Arrhythmias were observed in only one patient. Heart transplantation was performed in 25%–31.8% patients from 4 to 8 years old, with some developing secondary neurological symptoms. Most patients lost ambulation, required ventilation support, and exhibited orthopedic involvement. Overall mortality ranged from 18.25% to 50%, with sudden death being the most reported cause. The p. Pro209Leu variant in BAG3 is associated with progressive neurological and cardiac involvement, leading to a poor prognosis. Repeated cardiac screening is recommended in these patients and conversely, neurological progression should be monitored after transplantation in patients initially presenting with isolated RCM.

## 1 Introduction

Childhood-onset cardiomyopathies are rare, with an incidence estimated at 1.3 per 100,000 children (Lipshultz et al., 2003), and severe, carrying a significant risk of morbidity and mortality. The etiologies are diverse, ranging from sarcomeric genetic variants to metabolic diseases (Lipshultz et al., 2019). Among the rarer causes, neuromuscular diseases (NMDs) represent 8% of cases and are associated with a poorer prognosis (Towbin et al., 2006). One subgroup of NMDs is myofibrillar myopathies (MFMs), characterized by the disintegration of the myofiber Z-disk, followed by the accumulation of myofibrillar products and ectopic expression of multiple proteins (Selcen, 2011). These processes lead to structural and functional impairments of the Z-disk in both skeletal and cardiac muscles. The clinical features of MFMs are variable and include progressive muscle weakness, peripheral neuropathy, and cardiomyopathy, which typically present after the fourth decade and progress slowly (Silvestri et al., 2018).

*BAG3*-related myofibrillar myopathy is an exception, with clinical manifestations beginning in childhood. The first description of this condition was in 2009, when Selcen et al. described three unrelated children with MFM, each carrying a single nucleotide polymorphism in exon three of the *BAG3* gene, leading to the substitution of leucine by proline at position 209 (P209L) (Selcen et al., 2009). The three children exhibited progressive and severe muscle weakness during childhood, respiratory insufficiency, and a mixed restrictive/hypertrophic cardiomyopathy. Since this initial report, various case studies have been published, highlighting a clinical presentation characterized by both severe neurological and cardiac prognosis. Despite the increasing recognition of *BAG3*-related myofibrillar myopathies, the literature remains sparse, particularly regarding the cardiac phenotype as most studies focused on genetic and muscular aspects.

This study aims to provide a detailed cases serie of pediatric patients carrying the p. Pro209Leu BAG3 variant, focusing on their cardiac phenotype and clinical management, alongside a review of the literature.

## 2 Materials and methods

The retrospective study was conducted at hospital Necker-Enfants malades, Paris, and included patients diagnosed with myofibrillar myopathy and cardiomyopathy, carrying p. Pro209Leu variant in the BAG3 gene. Patient inclusion was based on active file data of 1,000 patients monitored for cardiomyopathies in the medical center per year, between 2012 and 2024.

The study adhered to the ethical standards of the institution and followed the guidelines established by the Declaration of Helsinki. This retrospective study was approved and recorded in the digital data processing registry of the APHP (Assistance Publique-Hôpitaux de Paris, protocol registration number: 20250225162956).

### 2.1 Clinical and biological data

Clinical data were collected from electronic medical records, including symptom onset, cardiac evaluations, biological data, outcomes and genetics data. The latest available data were preferred. All patient’s routine care management was performed by the same physician specialized in pediatric cardiomyopathies. Results were presented as medians with interquartile ranges [IQR].

### 2.2 Echocardiographic data

Echocardiographic assessments were performed for all patients by the same operator, experienced in pediatric cardiomyopathies. Data were measured according to guidelines from the American Society of Echocardiography (ASE) and the biplane method of discs was preferred for ejection fraction (EF) quantification (Lang et al., 2015). Measurements were expressed as Z-scores or adjusted for body surface area and were compared to the normal pediatric population data (Pettersen et al., 2008).

### 2.3 Cardiac magnetic resonance imaging (CMR) data

The left and right end-systolic and end-diastolic ventricular volumes normalized for body surface area (LVESVI, LVEDVI, RVESVI, RVEDVI) and LV/RV ejection fractions (LVEF/RVEF) were determined using Medis (Medis Suite 4.0.70.4). Left atrium and right atrium volumes were measured and indexed by body surface area (BSA) and compared to reference ranges from Voges et al. (Voges et al., 2021). Description of LGE location was based on American Heart Association (AHA) 17 segment model (Cerqueira et al., 2002). Fibrosis evaluation and strain analysis methods are reported in supplementary data.

### 2.4 Literature review

A literature review was conducted up to September 2025 using PubMed. We used the MESH-terms “myofibrillar myopathy,” “*BAG3*,” and “*BAG3*-related myopathy” to identify all relevant articles published in English. Clinical characteristics and outcomes of all reported patients were extracted and summarized. Patients in our series have been previously reported in Fernández-Eulate et al., 2025, updated clinical and follow-up data were collected for the purpose of this analysis.

## 3 Results

We included three male patients who had a cardiomyopathy associated with myofibrillar myopathy and carrying the p. Pro209Leu missense variant in the BAG3 gene.

### 3.1 Clinical, biomarker, and electrocardiographic characteristics

The first clinical signs appeared at a median age of 6 years [5.5–7] old, primarily with neurological symptoms, including progressive motor weakness and delayed motor milestones (Table 1). Cardiac symptoms manifested at a median age of 13 years [12–13]. Patient 1 initially presented with severe right-heart congestion symptoms, including exudative enteropathy, followed by left-heart congestion with marked dyspnea at rest (NYHA 4), leading to the diagnosis of restrictive cardiomyopathy (RCM). The diagnosis of myofibrillar myopathy due to a variant in *BAG3* was suggested by the cardiologist given the clinical presentation. Patient 2 experienced dyspnea during physical activity (NYHA 2), which progressively worsened to symptoms of right-sided heart failure over time. Patient 3, who was identified through presymptomatic cardiac screening on neurologist’s request, developed symptoms gradually during follow-up.

**TABLE 1:** Clinical data of patients.

	Patient 1	Patient 2	Patient 3	All
Onset				
Age at neurological onset (y)	5	8	6	6 (5.5–7)
Age at cardiac onset (y)	13	11	13	13 (12–13)
Time between neurological and cardiac onset (y)	8	3	7	7 (5–7.5)
Cardiac phenotype				
Clinical data at first exam				
NYHA	4	2	1	2 (1.5–3)
Right heart failure symptoms	+	+	-	2/3
Electrocardiogram and rhythmic events				
Biatrial electrical overload, incomplete RBBB and repolarization abnormalities	+	+	+	All
QTc (ms)	380	405	450	405 (392.5–427.5)
Ectopic atrial tachycardia	+	+	-	2/3
Atrial flutter	-	+	-	1/3
Biological data				
NT pro BNP (ng/mL) at first exam NR < 125	1990	3,580	856	1990 (1,423–2,785)
NT pro BNP (ng/mL) at last exam	7,410	735	896	896 (815.5–4,153)
CPK (IU/L) at first exam NR < 300	519	787	640	640 (579.5–713.5)
CPK (IU/L) at last exam	936	2,155	151	936 (543.5–1,545.5)
Echocardiography data				
Left atrial volume (mL/m^2^) NR < 34 mL/m^2^	50	53	37	50 (43.5–51.5)
Right atrial volume (mL/m^2^) NR < 32 mL/m^2^	50	104	45	50 (47.5–77)
IVSd (mm) NR < 11 mm	13	8	11	11 (9.5–12)
IVSd z-score	+3	+0.9	+2.4	+2.4 (1.65–2.7)
Mitral E/A ratio	3	4.7	3	3 (3–3.85)
Lateral mitral E’ (cm/s)	10	16	15	15 (12.5–15.5)
Medial mitral E’ (cm/s)	6	10	13	10 (8–11.5)
Lateral mitral E/e’	4.8	5.3	4.8	4,8 (4.8–5.05)
LVEF (%) NR > 55%	65	72	60	65 (63–69)
PAH	+	+	+	All
CMR				
CMR’s age (y)	13	13	10	13 (11.5–13)
LVMass (g/m^2^) NR 62 ± 11	91	43	37	43 (40–67)
LVEDVi (mL/m^2^) NR 79 ± 15	60	58	46	58 (52–59)
LVEF (%) NR 60 ± 7	56	60	53	56 (54.5–58)
RVEDVi (mL/m^2^) NR 83 ± 13	56	48	48	48 (48–52)
RVEF (%) NR 66 ± 7	56	60	43	56 (49.5–58)
RV free wall strain (-%) NR −29.3 (−30.6 to −28.1)	−34	−24	−35	−34 (−35.5–−29)
LAVi (mL/m2) NR 13.6 ± 4.9–34.1 ± 9.1	47	38	53	47 (42.5–50)
RAVi (mL/m2) NR 19.2 ± 6.8–34.2 ± 9.6	71	157	89	89 (80–123)
LA strain total (%) NR > 37%	34	24	28	28 (26–31)
LA EF total (%) NR > 59%	49	28	58	49 (28–58)
RA EF total (%) NR 48 ± 8	6.2	16.5	32	16.5 (11.35–24.25)
LGE	LV and Bi atrial	Bi atrial	LV	All
Follow-up				
Last follow up status (*age*)	Death (14)	Alive (20)	Death (18)	

Values are expressed as median (IQR); + present; - absent; Echocardiography z-score according to (Pettersen et al., 2008); Z-scores > +2 SD, indicate hypertrophy; normal range of CMR, according to Kawel-Boehm et al. (2020), Kowallick et al. (2014) and Krittanawong et al. (2023). CMR, cardiac magnetic resonance imaging; CPK, creatine phosphokinase; EF, ejection fraction; IVSd, interventricular septum in diastole; LA, left atrial; LAVi, Left atrial volume index; LGE, late gadolinium enhancement; LV, left ventricle; LVEF, left ventricular ejection fraction; LVMass, Left ventricular mass; LVEDVi, Indexed Left Ventricular End-Diastolic Volume; NR, normal range; NTproBNP, N-terminal pro B-type natriuretic peptide; NYHA, the New York Heart Association Functional Classification (New York Heart Association and Criteria Committee, 1994); PAH, pulmonary arterial hypertension; QTc, corrected QT, interval; RBBB, right bundle branch block; RA, right atrial; RAVi, Right atrial volume index; RV, right ventricle; RVESVi, Indexed Right Ventricular End-Systolic Volume; RVEF, right ventricular ejection fraction; (y), Years.

NT-proBNP levels were moderately elevated in all three patients and decreased with diuretic treatment, except for patient 1, who continued to show severe symptoms from the onset of care. Additionally, creatine kinase (CK) levels were mildly elevated in all patients, reflecting muscle damage associated with myofibrillar myopathy.

**FIGURE 1:**
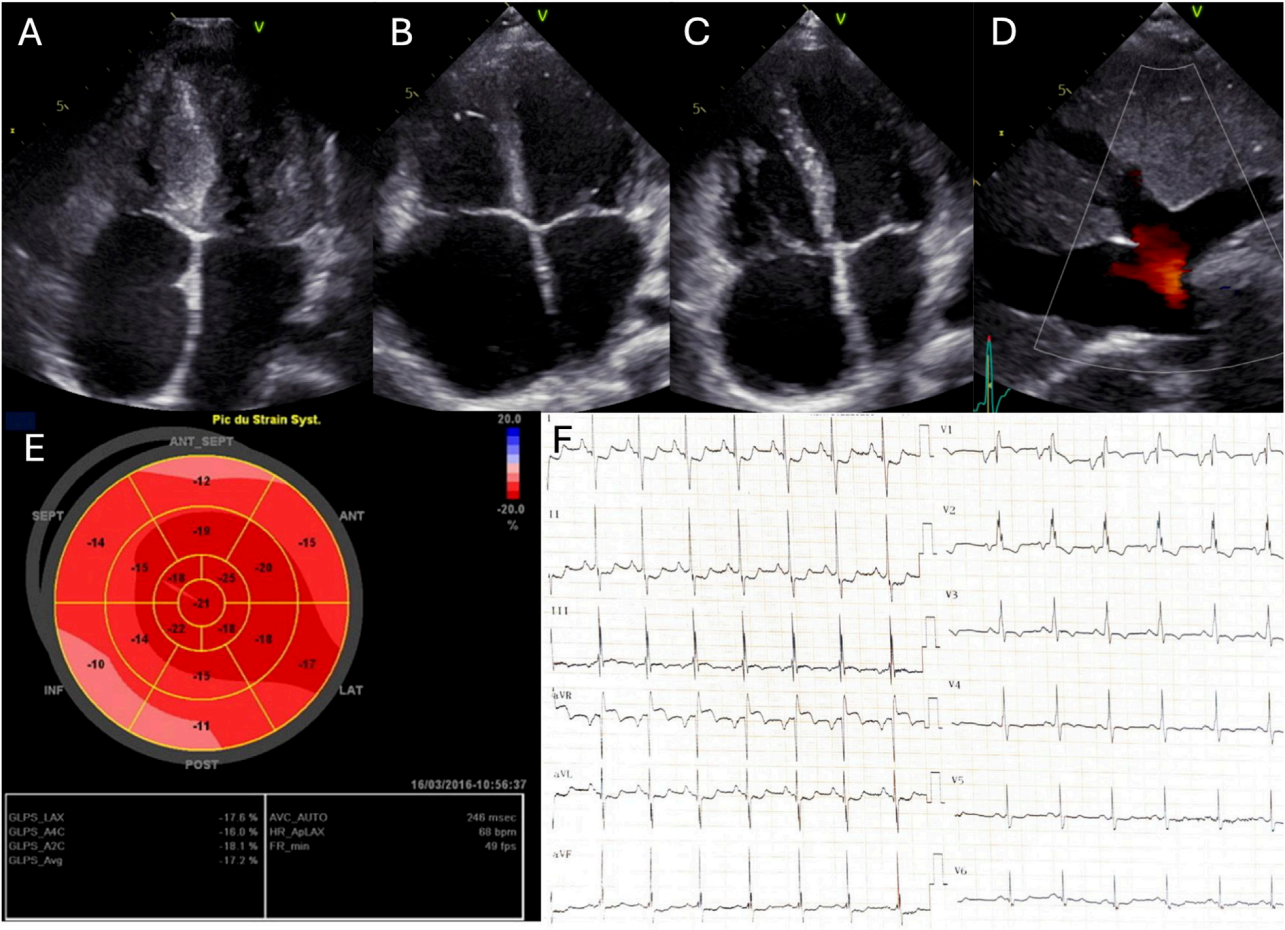
Echocardiography and electrocardiogram. A, B, C Four-chamber echocardiogram at end-diastole of patient 1 **(A)**, patient 2 **(B)** and patient 3 **(C)**; **(D)** Subcostal echocardiographic view of patient 2; **(E)** LV strain longitudinal of patient 3; **(F)** Electrocardiogram of patient 1.

Electrocardiograms (ECGs) showed bi-atrial hypertrophy and repolarization abnormalities including ST depression secondary to subendocardial ischemia, in all patients (Figure 1F). In addition, we observed a borderline QT prolongation (450 m) in patient 3, though no ventricular arrhythmias were detected, and there was no family history of such events. 24-h Holter-ECG revealed ectopic atrial tachycardia in patient 1 whereas symptomatic typical atrial flutter and then polymorphic atrial tachycardia was clinically documented in patient 2.

### 3.2 Echocardiography and CMR

All patients exhibited restrictive cardiomyopathy, with ventricular hypertrophy in two patients (patient 1 and 3) (Table 1; Figure 1). Echocardiography revealed restrictive physiology of the left ventricle, reduced diastolic compliance, bi-atrial enlargement and normal left ventricular systolic function. Right-sided filling pressures were elevated in all patients. Patient 2 and three displayed right-dominant restrictive cardiomyopathies, with significant right atrial dilation.

All patients underwent CMR. Both left and right ventricular volumes were normal, with preserved systolic function. Left ventricular mass was slightly elevated in Patient 1. Right and left atrial volumes were significantly increased in all patients with a predominant enlargement of the right atrium. No intracavitary thrombi were detected.

**FIGURE 2:**
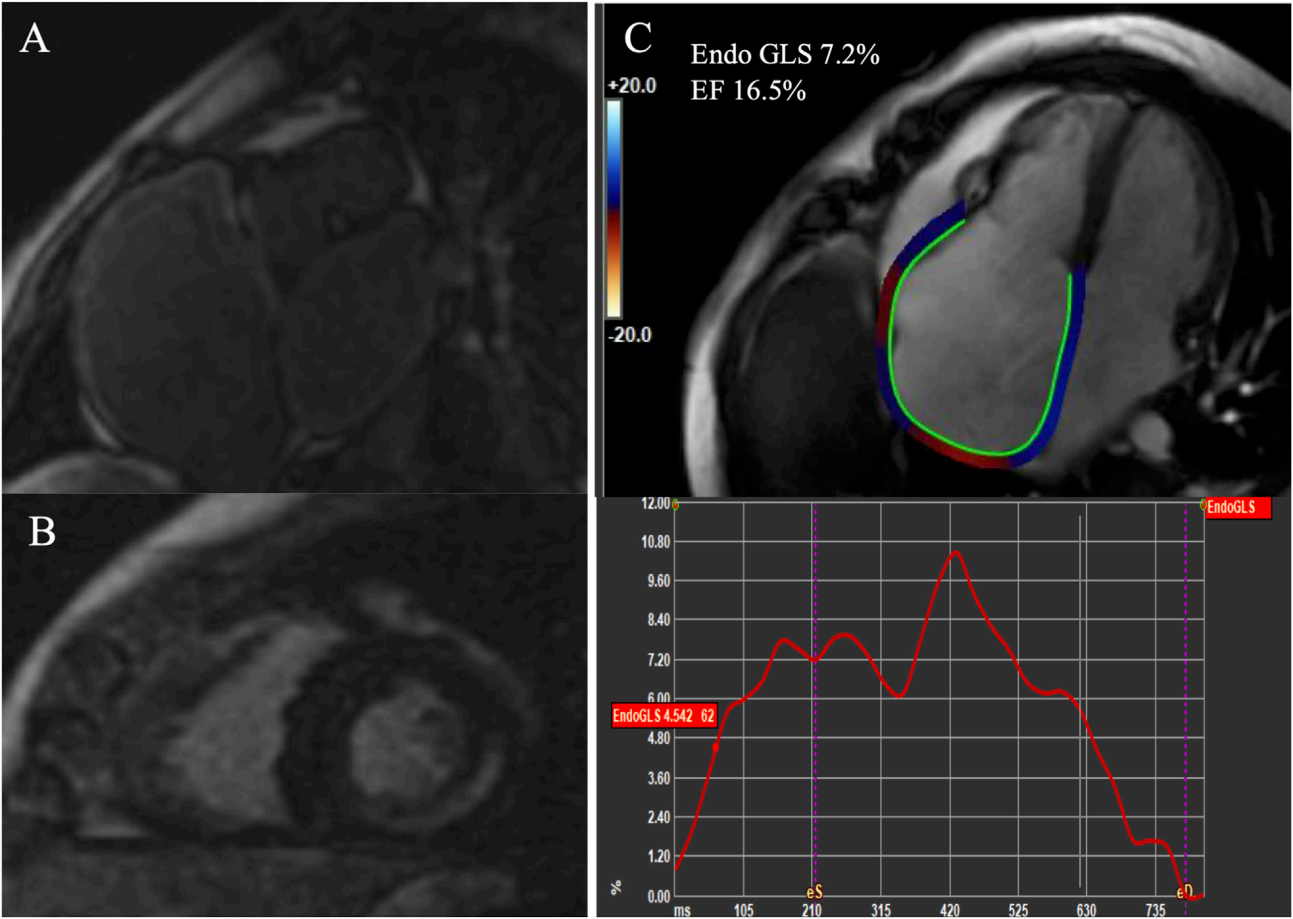
CMR data **(A)** Bi atrial LGE; **(B)** Left ventricle LGE; **(C)** RA strain of patient 2. LGE Late gadolinium enhancement, RA right atrial.

Left ventricle fibrosis was found in patient 1 and 3, involving the basal and mid-inferolateral segments in patient 1, and the basal inferior and basal inferolateral segments in patient 3 (Figure 2). Bi-atrial fibrosis was present in patient 1 and 2. The LA strain was reduced in all patients with a greater reduction in patient 2 and patient 3. Moreover, the LA EF was severely reduced in patient 1 and 2, mildly reduced in the patient three probably due to the absence of fibrosis at atrial level. The RA EF was severely reduced in the patient 1 and patient 2 but less impaired in patient 3.

### 3.3 Genetic diagnosis

In the three patients, genetic diagnosis was made at a median of 3 years [3–4.5] after the onset of symptoms, by an exome for two of them and a targeted genetic analysis of *BAG3* for patient three thanks to the medical staff knowledge of this particular *BAG3* cardiomyopathy. The same heterozygous missense variant in the *BAG3* (c.626C>T in exon 3, p. Pro209Leu) was identified. This variant is absent from the gnomAD database and is classified as pathogenic (ACMG class 5) (Richards et al., 2015). The three patients were unrelated. Genetic and phenotypic familial analysis confirmed that variants were *de novo*.

### 3.4 Management and follow-up

The three patients received a combination of spironolactone and furosemide. Dapagliflozine was added to diuretics for patient 2 because of persistent right sided congestion. All patients were on beta-blockers in order to lower heart rate to improve ventricle filling and to prevent arrhythmia. Patient 2 who had typical atrial flutter required anticoagulation, antiarrhythmic treatment (sotalol), and then underwent catheter ablation (cavotricuspid isthmus ablation). During follow-up, he experienced recurrent atrial arrhythmias, including polymorphic atrial tachycardia requiring further catheter ablation (three ectopic foci targeted in the right atrium). Two patients (patient 1 and 2) participated in an experimental trial (approved by the local ethical committee) with metformin (2 g per day), which has shown potential benefits in animal models of *BAG3*-related myofibrillar myopathy (Ruparelia et al., 2021), though its efficacy in humans remains uncertain. However, no significant improvement in cardiac or neuromuscular symptoms was observed. Heart transplantation was considered for patient 1 but was contraindicated due to advanced respiratory failure, as assessed by polysomnography and electroneuromyography (ENMG) and requiring respiratory support.

Generalized muscle weakness and gait disturbances were observed in all patients. Ambulation was lost in two patient (patient 1 and 3) by the ages of 13 and 12, respectively. None of the patients displayed cognitive impairment. Patient 1 and 3 developed restrictive respiratory failure, requiring continuous non-invasive ventilation. Patient 3 ended up needing tracheostomy. Additionally, all patients had associated musculoskeletal issues, including scoliosis, which required spinal fusion surgery at age 15 for Patient 3, and bilateral ankle arthrodesis at age 16 for Patient 2. Both patients underwent surgery under general anesthesia without any cardiac complications.

Heart failure episodes requiring hospitalization occurred in all patients, though predominantly in patient 1, who had a particularly severe presentation from diagnosis. In Patient 2 and 3, cardiac clinical symptoms were well controlled with diuretics treatment, likely also due to exercise limitation imposed by the neuromuscular condition.

Two out of three patients died. Patient 1, who had the most severe clinical presentation, succumbed to respiratory failure associated with heart failure at age 14, despite maximal medical therapy, 1 year after being diagnosed with restrictive cardiomyopathy. Patient 3 died of a ventilatory complication at age 18 but was stable as for the cardiac involvement of the disease. The only survivor, patient 2, is still ambulatory and does not require ventilatory support at age 20.

## 4 Discussion

The p. Pro209Leu pathogenic variation in the BAG3 gene is associated with childhood onset myofibrillar myopathy and severe cardiomyopathy. We describe the cardiac phenotype of three patients with *de novo* variant. All three patients had a restrictive cardiomyopathy with bi atrial dilation, no or mild cardiac hypertrophy and myocardial fibrosis. Patients mainly suffered from right-heart congestion. Significant supraventricular arrhythmias were observed in two patients.

A review of reported cases carrying this pathogenic variant revealed findings consistent with our cohort (Table 2, supplementary data). Recent data from Fernandez-Eulate et al. were presented in a separate column due to partial overlap with previously published patients. Neurological symptoms were the principal mode of disease discovery (68.2%–81.2%). Gait disturbances and muscle weakness were the most common initial neurological symptoms and over time, all patients developed neurological symptoms of an axonal neuropathy. However, cardiac symptoms (chest pain, heart murmur and heart failure) were the first reported signs and appears before neurological signs in at least 5 patients (Jaffer et al., 2012; Konersman et al., 2015; Schänzer et al., 2018; Scarpini et al., 2021).

**TABLE 2:** Literature review of patients carrying the BAG3 p.Pro209Leu variant.

​	Review of literature^a^	Fernandez-Eulate et al., 2025^b^
Number of patients	22	16
Male	11 (50)	10 (62.5)
First symptoms		
- Age at onset of first symptoms (y)^b^	8 [4.5–10.5]	7.8 ± 3.4
- Neuromuscular	15 (68.2)	13 (81.2)
- Cardiac	5 (22.7)	3 (18.7)
- Orthopedic	4 (18.2)	0
Clinical data		
Cardiac involvement	17 (77.3)	12 (75)
- Onset age (y)	11 [8.25–12.75]	NA
- RCM	15 (68)	12 (75)
with hypertrophy	6 (27.3)	NA
Onset age (y)^c^	12 [9.5–13]	NA
- HCM	1 (4.5)	0
Onset age (y)	11	-
- Long or borderline QT	3 (13.6)	0
- Arrhythmia	1 (4.5)	0
- HTx	7 (31.8)	4 (25)
Onset age (y)	13 [10.5–13.5]	From 8 to 14
Neuro-muscular involvement	22 (100)	16 (100)
- LoA	7 (31.8)	10 (62.5)
- Onset age of LoA (y)	15 [14–16.75]	18.6 ± 5.9
Respiratory involvement	18 (81.8)	14 (93.3)
- Ventilation	9 (40.9)	11 (68.8)
Orthopedic involvement	18 (81.8)	(93.8)
Outcomes		
Age at last evaluation	15.5 [14–19.75]	21.5 ± 8.6
Time from onset of symptoms (y)^c^	10 [5.25–11.75]	13.8 ± 7.4
Deceased	4 (18.25)	8 (50)
- Age at death (y)	14 [12–16.25]	22.5 ± 9.6
- Sudden death	3 (13.6)	5 (31.3)
- Respiratory insufficiency	1 (4.5)	1 (6.3)
- Heart failure	0	1 (6.3)
- Unknown	0	1 (6.3)

Values are expressed as n (%), mean ± SD, median [IQR], HCM, hypertrophic cardiomyopathy; NA, not available; RCM, restrictive cardiomyopathy; (y), years; (−), not applicable.

^a^ Review of literature available on supplementary data, from references: Selcen et al., 2009; Odgerel et al., 2010; Lee HC, et al., 2012; Jaffer et al., 2012; Kostera-Pruszczyk et al., 2015; Konersman et al., 2015; D’avila et al., 2016; Kim et al., 2018; Noury et al., 2018; Schänzer et al., 2018; Andersen et al., 2018; Malatesta et al., 2020; Scarpini et al., 2021; Xu et al., 2021; Akaba et al., 2022; Angelini et al., 2023.

^b^ Fernandez-Eulate et al., 2025: the study population included some patients previously reported in the articles listed above: Selcen et al., 2009; Odgerel et al., 2010; Lee HC et al., 2012; Jaffer et al., 2012; Kostera et al., 2015; Konersman et al., 2015; Kim et al., 2018; Noury et al., 2018; Schänzer et al., 2018; Andersen et al., 2018; Malatesta et al., 2020; Scarpini et al., 2021; Xu et al., 2021; Akaba et al., 2022; De Fuenmayor-Fernández de la Hoz et al., 2024; Zhan et al., 2022; Angelini et al., 2023.

^c^ Median values were calculated based on the number of available observations.

Cardiac involvement affected 75%–77.3% of these patients. Early-onset cardiomyopathy was the most frequently reported feature, exhibiting a restrictive pattern in 68%–75% of cases and associated to ventricular hypertrophy in six cases. One patient had mild LV wall thickening, raising the possibility of restrictive phenotype seen at an early stage (D’avila et al., 2016). In our literature review, five patients had no cardiac anomalies despite similar neurological, respiratory, and orthopedic symptoms (Andersen et al., 2018; Kim et al., 2018; Noury et al., 2018; Malatesta et al., 2020; Akaba et al., 2022), suggesting a variable expressivity of the cardiac phenotype. A prolonged or borderline QT interval was observed in three patients overall (Lee et al., 2012; Kostera-Pruszczyk et al., 2015; Xu et al., 2021). Conduction disturbances and arrhythmias were reported in one case (D’Avila et al., 2016). This incidence might have been underestimated in prior studies which focused on the description of neuromuscular presentation. We observed bi-atrial fibrosis on CMR in both patients with supraventricular arrhythmias. Therefore, our case series highlights that rhythm monitoring is essential in these patients for an early diagnosis of atrial arrhythmias and the prevention of potential thromboembolic complications.

Respiratory involvement affected 81.8%–93.3% of patient with ventilation support required for most of them. Heart transplantation was performed in 25%–31.8% of patients, with extra-cardiac manifestations occuring post-transplant in three cases (Jaffer et al., 2012; Konersman et al., 2015; Schänzer et al., 2018). Despite heart transplantation, three patients died: one boy at 15 years old, 2 years after transplant, and two additional patients more than 10 years post-transplant (Odgerel et al., 2010; Fernández-Eulate et al., 2025). In our literature review, six transplanted patients were a live at last follow-up, with a median post-transplant survival of 3.5 [2.25–9.25] years but had respiratory and orthopedic involvements. We believe that early referral to cardiologist can delay and improve heart transplant prognosis. Overall mortality was high, ranged from 18.25% to 50% of patients dying; the most frequent cause was sudden death, raising the possibility of underlying ventricular arrhythmias. Survivors had significant morbidity, including loss of ambulation, dependence on ventilation, and scoliosis. Notably, two patients survived to age 26, both with loss of ambulation, and one without cardiac involvement (D’Avila et al., 2016; Andersen et al., 2018).

Cardiac diagnosis delays in these patients are often prolonged due to the rarity of the condition and the absence of systematic cardiac evaluations in myopathies. However, the latest research supports routine echocardiographic assessments at diagnosis and yearly thereafter for patients with any form of myofibrillar myopathies (Silvestri et al., 2018). The inclusion of *BAG3* in the gene panel testing for myofibrillar and inclusion body myopathies could improve the diagnosis yield of this condition and have already been implemented in France by the neuromuscular rare disease network, FILNEMUS, which has standardized national clinical practices (Krahn et al., 2019). In patients of atypical myopathy with negative panel results, expanded diagnostic approaches such as large NGS panels, WES (whole exome sequencing), WGS (whole genome sequencing), or RNA sequencing may also be useful and has also been recommended in France in order to improve diagnostic precision.

*BAG3* pathogenic variations are associated to diverse neurological and cardiovascular diseases, including isolated cardiomyopathy in adults (OMIM 613881) and myofibrillar myopathy (OMIM 612954). These diseases are transmitted in an autosomal dominant manner, but most cases occur *de novo*, particularly in myofibrillar myopathy. Among cardiac diseases, DCM is the most frequent but *BAG3* variants represent only 0.3% of all DCM (Mazzarotto et al., 2020). A study on 129 *BAG3* variants carriers, found that 68.4% of patients had DCM, with a mean diagnosis age of 36.9 years and poor response to standard therapies (Domínguez et al., 2018). In contrast, cardiac involvement appeared earlier and was more severe in our cohort and in patients carrying the missense p. Pro209Leu variation. The *BAG3* gene encodes Bcl-2 associated-athanogene-3, a multifunctional protein acting as a co-chaperone for heat shock proteins (HSP) and promoting degradation via autophagy (Martin et al., 2021). It regulates critical processes in the heart and other tissues, including sarcomere stabilization (Martin et al., 2021), nuclear envelope integrity (Gupta et al., 2019), autophagy activation and apoptosis inhibition (Feldman et al., 2014). It is primarily expressed in skeletal muscle and cardiomyocytes, essential for cardiac sarcomere maintenance through protein turnover. *BAG3* is a key candidate gene in dilated cardiomyopathy (DCM) cases, including peripartum DCM, cardiotoxicity from chemotherapy, and myocarditis (Mazzarotto et al., 2020), and is linked to tumor growth (Liu et al., 2009; Colvin et al., 2014). Recent studies suggest a strong association between *BAG3* variants and cardiac fibrotic remodeling through TGF-β dysregulation, which may contribute to the development of cardiomyopathies (Frangogiannis, 2022; Wang et al., 2025). The fibrogenic response induce by loss of *BAG3* is particularly relevant in the atria, where fibroblasts are already more responsive to TGF-β activation, suggesting that *BAG3* variants might preferentially exacerbate atrial fibrosis rather than ventricular fibrosis (Wang et al., 2025). This is consistent with our clinical observations of the three patients carrying BAG3 p. Pro209Leu variant, who present atrial dysfunction with myocardial fibrosis and atrial arrhythmias.

Most pathogenic *BAG3* variants are deletions or truncations in the BAG or WW domains causing DCM by a loss-of-function mechanism (Qu et al., 2022). The p. Pro209Leu variation is one of the only reported missense pathogenic variation of BAG3. This variation is located in the second IPV domain. It has been showed in zebrafish model that this variation is responsible of the formation of aggregates with wild-type *BAG3*, leading to protein insufficiency and can be considered as a toxic gain-of-function variant (Ruparelia et al., 2014). This different pathophysiological mechanism probably explains the severe and early-onset phenotype observed in patients carrying this variation.

Autophagy stimulation could enhance aggregate clearance, offering a potential therapeutic strategy for patients with the BAG3-Pro209Leu variant (Schänzer et al., 2018). In 2021, metformin was tested and induced reduction of protein aggregates in zebrafish and human myoblasts but was also able to rescue the fiber disintegration and swimming deficit observed in fish (Ruparelia et al., 2021). Another therapeutic approach involves gene therapies. In 2024, Shin et al. developed personalized allele-specific CRISPR-Cas9 strategies to selectively inactivate the mutant allele in patient-derived induced pluripotent stem cells (iPSCs), offering promising therapeutic potential (Shin et al., 2024).

## 5 Conclusion

Our study reinforces the early-onset and severe cardiomyopathy associated to the p. Pro209Leu *BAG3* variation, underscoring cardiac involvement as the major determinant of prognosis. As the initial presentation often occurs through neurological manifestations, cardiomyopathy is frequently diagnosed at a later stage and by non-specialized cardiologists. Establishing the genetic diagnosis may facilitate earlier referral to specialized cardiac care, with the potential to delay disease progression and optimize preparation for heart transplantation. Anticipatory management is crucial to ensure transplantation under the best possible clinical and psychological conditions, thereby improving overall patient outcomes.

## Data Availability

The datasets presented in this study can be found in online repositories. The names of the repository/repositories and accession number(s) can be found below: https://www.ncbi.nlm.nih.gov/clinvar/, SCV004031322.1.

## Supplementary material

SUPPLEMENTARY DATA 1 Protocols for CMR image acquisition and analysis.

SUPPLEMENTARY DATA 2 Literature review.
