# Correction: *BAG3*-related myofibrillar myopathy: focus on its cardiac involvement

**DOI:** 10.3389/fgene.2026.1786473

**Published:** 2026-02-09

**Authors:** Elise Daire, Elena Panaioli, Cyril Gitiaux, Catherine Gardin, Victor Waldmann, Damien Bonnet, Karim Wahbi, Diala Khraiche

**Affiliations:** 1 Pediatric Cardiology Department, Amiens University Hospital and Laboratory EA4666 Hematim, University of Picardie–Jules Verne, Amiens, France; 2 M3C-Necker, Congenital and Pediatric Cardiology Department, Necker Enfants malades University Hospital, APHP, Paris, France; 3 Reference Centre for Neuromuscular Diseases, Necker-Enfants malades Hospital, APHP, Paris, France; 4 Cardiology Departement, Laval Hospital, Laval, France; 5 University Paris Cité, Paris, France; 6 APHP, Cochin Hospital, Cardiology Department, Centre de Référence de Pathologie Neuromusculaire, Paris, France

**Keywords:** cardiomyopathy, pediatric, BAG3, Bcl-2-associated athanogene 3, myofibrillar myopathy, restrictive cardiomyopathy

“DeFuenmayor-Fernández de la Hoz et al., doi 10.1007/s00415-023-12039-9” and “Zhan et al., 2022 doi: 10.1097/MD.0000000000028484” were not cited in the article. The citation has now been inserted in the caption of Table 2 and should read:

**TABLE 2:** Literature review of patients carrying the BAG3 p.Pro209Leu variant.

​	Review of literature^a^	Fernandez-Eulate et al., 2025^b^
Number of patients	22	16
Male	11 (50)	10 (62.5)
First symptoms		
- Age at onset of first symptoms (y)^b^	8 [4.5–10.5]	7.8 ± 3.4
- Neuromuscular	15 (68.2)	13 (81.2)
- Cardiac	5 (22.7)	3 (18.7)
- Orthopedic	4 (18.2)	0
Clinical data		
Cardiac involvement	17 (77.3)	12 (75)
- Onset age (y)	11 [8.25–12.75]	NA
- RCM	15 (68)	12 (75)
with hypertrophy	6 (27.3)	NA
Onset age (y)^c^	12 [9.5–13]	NA
- HCM	1 (4.5)	0
Onset age (y)	11	-
- Long or borderline QT	3 (13.6)	0
- Arrhythmia	1 (4.5)	0
- HTx	7 (31.8)	4 (25)
Onset age (y)	13 [10.5–13.5]	From 8 to 14
Neuro-muscular involvement	22 (100)	16 (100)
- LoA	7 (31.8)	10 (62.5)
- Onset age of LoA (y)	15 [14–16.75]	18.6 ± 5.9
Respiratory involvement	18 (81.8)	14 (93.3)
- Ventilation	9 (40.9)	11 (68.8)
Orthopedic involvement	18 (81.8)	(93.8)
Outcomes		
Age at last evaluation	15.5 [14–19.75]	21.5 ± 8.6
Time from onset of symptoms (y)^c^	10 [5.25–11.75]	13.8 ± 7.4
Deceased	4 (18.25)	8 (50)
- Age at death (y)	14 [12–16.25]	22.5 ± 9.6
- Sudden death	3 (13.6)	5 (31.3)
- Respiratory insufficiency	1 (4.5)	1 (6.3)
- Heart failure	0	1 (6.3)
- Unknown	0	1 (6.3)

“Table 2 Literature review of patients carrying the BAG3 p.Pro209Leu variant.

Values are expressed as n (%), mean ± SD, median [IQR], HCM, Hypertrophic cardiomyopathy; HTx Heart transplantation; LoA Loss of Ambulation; NA, not available; RCM, restrictive cardiomyopathy; (y), years; (−), Not applicable. a Review of literature available on supplementary data, from references: Selcen et al., 2009; Odgerel et al., 2010; Lee HC et al., 2012; Jaffer et al., 2012; Kostera et al., 2015; Konersman et al., 2015; D’avila et al., 2016; Kim et al., 2018; Noury et al., 2018; Schänzer et al., 2018; Andersen et al., 2018; Malatesta et al., 2020; Scarpini et al., 2021; Xu et al., 2021; Akaba et al., 2022; Angelini et al., 2023; b Fernandez-Eulate et al., 2025: the study population included some patients previously reported in the articles listed above: Selcen et al., 2009; Odgerel et al., 2010; Lee HC et al., 2012; Jaffer et al., 2012; Kostera et al., 2015; Konersman et al., 2015; Kim et al., 2018; Noury et al., 2018; Schänzer et al., 2018; Andersen et al., 2018; Malatesta et al., 2020; Scarpini et al., 2021; Xu et al., 2021; Akaba et al., 2022; De Fuenmayor-Fernández de la Hoz et al., 2024; Zhan et al., 2022; Angelini et al., 2023c Median values were calculated based on the number of available observations.”

There was a mistake in Table 2 as published. The literature review included data from Fernandez-Eulate et al., 2025. However, this article, which included patients from previous publications, was inadvertently counted as a primary study, leading to duplicate inclusion of some cases and an overestimation of the total number of included studies. We have now carefully reassessed the literature and corrected Table 2. Recent data from Fernandez-Eulate et al. will be presented in a separate column due to partial overlap with previously published patients already included in our literature review, as well as the associated numerical data. The corrected Table 2 appears below.

There was a mistake in the caption of Table 2 as published. The caption of Table 2 contained an error in the published article. It has been revised to clarify which previously published studies contributed patients to the cohort reported by Fernández-Eulate et al., 2025, in order to avoid any potential duplication. The corrected caption of Table 2 appears below.

File Supplementary Table 2 was erroneously published with the original version of this paper. The file has now been replaced. In the Supplementary material section, the following has been removed: “SUPPLEMENTARY DATA TABLE S2 Values are expressed as n (%), median[IQR], HCM hypertrophic cardiomyopathy; HTx heart transplantation; LoA Lost of ambulation; NA not available; NIV Non invasive ventilation; RCM restriictive cardiomyopathy; RCM-H restrictive cardiomyopathy with hypertrophy; y years”. The updated legend is included in the updated Supplementary Table 2.

In the abstract:

o “In the reviewed cases, cardiac involvement was present in 76.9% and diagnosed at an early age of 11 [8.2–12.7].” has been corrected to read: “In the reviewed cases, cardiac involvement was present in 75%–77.3% of patients and was diagnosed at an early age.”
o “Restrictive cardiomyopathy was the most prevalent phenotype (69.2%), followed by hypertrophic cardiomyopathy (5.1%) and rare long or borderline QT interval (7.7%).” has been corrected to read: “Restrictive cardiomyopathy was the most prevalent phenotype (68%–75%), whereas few patients exhibited hypertrophic cardiomyopathy or borderline QT interval.”
o “Heart transplantation was performed in 11 patients at 13 [10.5–13.5] years, with some developing secondary neurological symptoms.” has been corrected to read: “Heart transplantation was performed in 25%–31.8% patients from 4 to 8 years old, with some developing secondary neurological symptoms.”
o “Overall mortality was 30.7%, with sudden death being the most reported cause.” has been corrected to read: “Overall mortality ranged from 18.25% to 50%, with sudden death being the most reported cause.”

A correction has been made to section 2.4 Literature review:

“Clinical characteristics and outcomes of all reported patients were extracted and summarized.”

This has been corrected to read:

“Clinical characteristics and outcomes of all reported patients were extracted and summarized. Patients in our series have been previously reported in Fernández-Eulate et al., 2025, updated clinical and follow-up data were collected for the purpose of this analysis.”

A correction has been made to section 4 Discussion:

“A review of 39 cases carrying this pathogenic variation from the literature reveals findings consistent with our cohort (Table 2). Neurological symptoms were the principal mode of disease discovery (74.3%). Gait disturbances and muscle weakness were the most common initial neurological symptoms and over time, all patients developed neurological symptoms of an axonal neuropathy. However, in eight patients, cardiac symptoms (chest pain, heart murmur and heart failure) were the first reported signs and appears before neurological signs (Jaffer et al., 2012; Konersman et al., 2015; Schänzer et al., 2018; Scarpini et al., 2021; Fernández-Eulate et al., 2025).

Cardiac involvement affected 76.9% of these patients. Early-onset cardiomyopathy was the most frequently reported feature, exhibiting a restrictive pattern in 69.2% of cases and associated to ventricular hypertrophy in six cases. Two patients had mild LV wall thinckening, raising the possibility of restrictive phenotype seen at an early stage. Nine patients had no cardiac anomalies despite similar neurological, respiratory, and orthopedic symptoms (Andersen et al., 2018; Kim et al., 2018; Noury et al., 2018; Malatesta et al., 2020; Akaba et al., 2022; Fernández-Eulate et al., 2025), suggesting a variable expressivity of the cardiac phenotype.”

This has been corrected to read: “A review of reported cases carrying this pathogenic variant revealed findings consistent with our cohort (Table 2, supplementary data). Recent data from Fernandez-Eulate et al. were presented in a separate column due to partial overlap with previously published patients. Neurological symptoms were the principal mode of disease discovery (68.2%–81.2%). Gait disturbances and muscle weakness were the most common initial neurological symptoms and over time, all patients developed neurological symptoms of an axonal neuropathy. However, cardiac symptoms (chest pain, heart murmur and heart failure) were the first reported signs and appears before neurological signs in at least 5 patients (Jaffer et al., 2012; Konersman et al., 2015; Schänzer et al., 2018; Scarpini et al., 2021).

Cardiac involvement affected 75%–77.3% of these patients. Early-onset cardiomyopathy was the most frequently reported feature, exhibiting a restrictive pattern in 68%–75% of cases and associated to ventricular hypertrophy in six cases. One patient had mild LV wall thickening, raising the possibility of restrictive phenotype seen at an early stage (D’avila et al., 2016). In our literature review, five patients had no cardiac anomalies despite similar neurological, respiratory, and orthopedic symptoms (Andersen et al., 2018; Kim et al., 2018; Noury et al., 2018; Malatesta et al., 2020; Akaba et al., 2022), suggesting a variable expressivity of the cardiac phenotype.”

“Respiratory involvement affected 84.6% of patient with ventilation support required for most of them. Heart transplantation was performed in 11 cases, with extra-cardiac manifestations occurring post-transplant in three cases (Jaffer et al., 2012; Konersman et al., 2015; Schänzer et al., 2018). Despite heart transplantation, three patients died: one boy at 15 years old, 2 years after transplant, and two additional patients more than 10 years post-transplant (Odgerel et al., 2010; Fernández-Eulate et al., 2025). Eight transplanted patients were a live at last follow-up, with a median post-transplant survival of 4 [2.75–10.25] years but had respiratory and orthopedic involvements. We believe that early referral to cardiologist can delay and improve heart transplant prognosis. Overall mortality was high, with 30.7% of patients dying; the most frequent cause was sudden death, raising the possibility of underlying ventricular arrhythmias.”

This has been corrected to read “Respiratory involvement affected 81.8%–93.3% of patient with ventilation support required for most of them. Heart transplantation was performed in 25%–31.8% of patients, with extra-cardiac manifestations occuring post-transplant in three cases (Jaffer et al., 2012; Konersman et al., 2015; Schänzer et al., 2018). Despite heart transplantation, three patients died: one boy at 15 years old, 2 years after transplant, and two additional patients more than 10 years post-transplant (Odgerel et al., 2010; Fernández-Eulate et al., 2025). In our literature review, six transplanted patients were a live at last follow-up, with a median post-transplant survival of 3.5 [2.25 – 9.25] years but had respiratory and orthopedic involvements. We believe that early referral to cardiologist can delay and improve heart transplant prognosis. Overall mortality was high, ranged from 18.25% to 50% of patients dying; the most frequent cause was sudden death, raising the possibility of underlying ventricular arrhythmias.”

The original article has been updated.

