# Supplementary material for: BAG3-related myofibrillar myopathy: focus on its cardiac involvement
Source: Front Genet. 2025 Nov 26;16:1636999. doi: 10.3389/fgene.2025.1636999 (PMC12688876; doi:10.3389/fgene.2025.1636999)
Supplement: Supplementary file 2 [file Table1.docx]

The left and right end-systolic and end-diastolic ventricular volumes normalized for body surface area (LVESVI, LVEDVI, RVESVI, RVEDVI) and LV/RV ejection fractions (LVEF/RVEF) were determined using Medis (Medis Suite 4.0.70.4). Left atrium and right atrium volumes were measured and indexed by body surface area (BSA) and compared to reference ranges from Voges et al (Voges *et al.*, 2021) . Description of LGE location was based on American Heart Association (AHA) 17 segment model (Cerqueira *et al.*, 2002) .

As far concerns the fibrosis evaluation for patients able to suspend respiration, breath-hold duration was 10 to 15 seconds, depending on the heart rate; otherwise, images were acquired using 3 signal averages. Subsequently, LGE images were obtained in the same long- and short-axis orientation as in the above-described balanced steady-state free precession images, ∼15-20 minutes after injection of 0.2 mmol/kg of gadolinium using a phase-sensitive inversion recovery spoiled gradient echo sequence.

All CMR parameters values were obtained based on semi-automatic methods using standard image analysis software. QStrain v4.0 (Medis Suite 4.0.70.4) was used for strain analysis. This software provides a rapid and simple procedure to manually locate mitral and tricuspid valves, semi-automatedly delineate RV, LA and RA endocardial and epicardial boundaries at end-diastole and present tracked boundaries at end-systole. The feature-tracking CMR-derived RV strain was obtained from four-chamber long-axis cine images (Yang *et al.*, 2024).

The feature tracking of the CMR-derived LA strain was obtained from two- and four-chamber long-axis cine images. LA endocardial contours were manually drawn on the end-diastolic and end-systolic images, excluding the LA appendage and pulmonary veins. The software automatically propagated the contours throughout the cardiac cycle. The quality of the automatic tracking was reviewed, and manual contour adjustments were made when necessary. Three aspects of LA strain were analyzed as previously described by Kowallick et al, 2014 (Kowallick *et al.*, 2014) : reservoir strain LARs, contractile strain LACs and conduit strain (Truong *et al.*, 2020). Moreover LA function has been evaluated by the estimation of LA EF total (%) (Truong *et al.*, 2020). RA volume was assessed using area-length method in the 4-chamber during various RA phase with the exclusion of RA appendage using Qmass (version 8.1, Medis). The RA total empty fraction, RA passive fraction, and RA active fraction were calculated using corresponding RA volumetric measurements. Manual adjustments were performed when necessary for optimal tracking (Li *et al.*, 2022). We have calculated right atria ejection fraction.

Cerqueira, M.D. et al. (2002) ‘Standardized myocardial segmentation and nomenclature for tomographic imaging of the heart. A statement for healthcare professionals from the Cardiac Imaging Committee of the Council on Clinical Cardiology of the American Heart Association’, Circulation, 105(4), pp. 539–542. Available at: https://doi.org/10.1161/hc0402.102975.

Kowallick, J.T. et al. (2014) ‘Quantification of left atrial strain and strain rate using Cardiovascular Magnetic Resonance myocardial feature tracking: a feasibility study’, Journal of Cardiovascular Magnetic Resonance: Official Journal of the Society for Cardiovascular Magnetic Resonance, 16(1), p. 60. Available at: https://doi.org/10.1186/s12968-014-0060-6.

Li, Y. et al. (2022) ‘Prognostic value of right atrial strain derived from cardiovascular magnetic resonance in non-ischemic dilated cardiomyopathy’, Journal of Cardiovascular Magnetic Resonance: Official Journal of the Society for Cardiovascular Magnetic Resonance, 24(1), p. 54. Available at: https://doi.org/10.1186/s12968-022-00894-w.

Truong, V.T. et al. (2020) ‘Normal left atrial strain and strain rate using cardiac magnetic resonance feature tracking in healthy volunteers’, European Heart Journal. Cardiovascular Imaging, 21(4), pp. 446–453. Available at: https://doi.org/10.1093/ehjci/jez157.

Voges, I. et al. (2021) ‘Normal values for paediatric atrial volumes assessed by steady-state free-precession cardiovascular magnetic resonance’, European Heart Journal, 42(Supplement_1), p. ehab724.1601. Available at: https://doi.org/10.1093/eurheartj/ehab724.1601.

Yang, W. et al. (2024) ‘Myocardial Strain Measurements Derived From MR Feature-Tracking’, JACC: Cardiovascular Imaging, 17(4), pp. 364–379. Available at: https://doi.org/10.1016/j.jcmg.2023.05.019.
